# ASO Author Reflections: Neoadjuvant Radiotherapy in Locally Advanced Esophageal Adenocarcinoma; Low-Dose Radiation, Low-Dose Expectations?

**DOI:** 10.1245/s10434-023-14885-3

**Published:** 2024-01-11

**Authors:** Styliani Mantziari, Hugo Teixeira Farinha, Guillaume Piessen, Markus Schäfer

**Affiliations:** 1 Department of Visceral Surgery, Lausanne University Hospital CHUV, Lausanne, Switzerland; 2 Faculty of Biology and Medicine, University of Lausanne UNIL, Lausanne, Switzerland; 3 Department of Digestive and Oncological Surgery, CHU Lille, Lille, France; 4 UMR9020-U1277 - CANTHER – Cancer Heterogeneity Plasticity and Resistance to Therapies, University of Lille, CNRS, Inserm, CHU Lille, Lille, France

## Past

Neoadjuvant treatment with either chemotherapy alone or chemoradiation (nCRT) has led to significant survival improvements in patients with esophageal cancer. However, large-scale data comparing chemoradiation protocols with each other remain scarce, resulting in high variability of treatment strategies in clinical practice. The 2012 CROSS trial^1^ established low-dose (41.4 Gy) radiotherapy with carboplatin-paclitaxel as the current standard of care in many centers, offering pathologic complete response (pCR) in 49% of patients with squamous cell cancer (SCC) and 23% with adenocarcinoma (AC).

## Present

Despite the compelling evidence suggesting a clear nCRT survival benefit compared with surgery alone,^1^ the ‘strict’ 41.4 Gy CROSS low-dose regimen has not been universally adopted worldwide. The reasons for that seem mostly related to local center and physician experience, as well as apprehension to undertreat bulky tumors with low-dose radiation. Currently, North American protocols recommend a neoadjuvant radiation dose between 41 Gy and 50.4 Gy,^2^ while European guidelines suggest 41.4 Gy for SCC and do not clearly specify the dose for AC, suggesting nevertheless the CROSS protocol.^3^ As for the chemotherapy backbone of neoadjuvant treatment, the carboplatin-paclitaxel regimen commonly used with the low-dose CROSS protocol was recently associated with higher rates of severe postoperative complications compared with FOLFOX.^4^ Current data suggest that low-dose radiation may yield lower rates of pCR in patients with locally advanced esophageal cancer.^5^ The difference may not be significant in SCC, but patients with AC treated with the low-dose regimen appear to have lower chances in obtaining pCR.^5^ Although patient survival is not necessarily correlated with pCR and the risk of added radiation-related morbidity (for doses > 55 Gy) needs to be considered, complete clinical/pathological response after CRT becomes an increasingly relevant outcome per se.

## Future

Major ongoing clinical trials are currently evaluating the watch-and-wait strategy for patients with complete response after nCRT, and their results may shape the future of esophageal cancer treatment. It is thus particularly important to be cautious of the risk of under-treating patients with AC with low-dose radiation, as they represent the predominant histological type in many regions worldwide.
